# Administration of *Akkermansia muciniphila* Ameliorates Dextran Sulfate Sodium-Induced Ulcerative Colitis in Mice

**DOI:** 10.3389/fmicb.2019.02259

**Published:** 2019-10-01

**Authors:** Xiaoyuan Bian, Wenrui Wu, Liya Yang, Longxian Lv, Qing Wang, Yating Li, Jianzhong Ye, Daiqiong Fang, Jingjing Wu, Xianwan Jiang, Ding Shi, Lanjuan Li

**Affiliations:** ^1^State Key Laboratory for Diagnosis and Treatment of Infectious Diseases, National Clinical Research Center for Infectious Diseases, The First Affiliated Hospital, College of Medicine, Zhejiang University, Hangzhou, China; ^2^Collaborative Innovation Center for Diagnosis and Treatment of Infectious Diseases, Zhejiang University, Hangzhou, China

**Keywords:** *Akkermansia muciniphila*, microbiota, IBD, DSS-induced colitis, metabolism

## Abstract

Inflammatory bowel diseases (IBDs) develop as a result of complex interactions among genes, innate immunity and environmental factors, which are related to the gut microbiota. Multiple clinical and animal data have shown that *Akkermansia muciniphila* is associated with a healthy mucosa. However, its precise role in colitis is currently unknown. Our study aimed to determine its protective effects and underlying mechanisms in a dextran sulfate sodium (DSS)-induced colitis mouse model. Twenty-four C57BL/6 male mice were administered *A. muciniphila* Muc^T^ or phosphate-buffered saline (PBS) once daily by oral gavage for 14 days. Colitis was induced by drinking 2% DSS from days 0 to 6, followed by 2 days of drinking normal water. Mice were weighed daily and then sacrificed on day 8. We found that *A. muciniphila* improved DSS-induced colitis, which was evidenced by reduced weight loss, colon length shortening and histopathology scores and enhanced barrier function. Serum and tissue levels of inflammatory cytokines and chemokines (TNF-α, IL1α, IL6, IL12A, MIP-1A, G-CSF, and KC) decreased as a result of *A. muciniphila* administration. Analysis of 16S rDNA sequences showed that *A. muciniphila* induced significant gut microbiota alterations. Furthermore, correlation analysis indicated that pro-inflammatory cytokines and other injury factors were negatively associated with *Verrucomicrobia*, *Akkermansia, Ruminococcaceae*, and *Rikenellaceae*, which were prominently abundant in *A. muciniphila*-treated mice. We confirmed that *A. muciniphila* treatment could ameliorate mucosal inflammation either via microbe-host interactions, which protect the gut barrier function and reduce the levels of inflammatory cytokines, or by improving the microbial community. Our findings suggest that *A. muciniphila* may be a potential probiotic agent for ameliorating colitis.

## Introduction

Inflammatory bowel diseases (IBDs), including ulcerative colitis (UC) and Crohn’s disease (CD), have developed into a global scourge with an increasing incidence and prevalence worldwide in the last 50 years (Kaplan, 2015; Parada Venegas et al., 2019). IBDs were convincingly shown to develop as a result of complex interactions among genes, innate immunity and environmental factors (Parada Venegas et al., 2019). Intriguingly, gut microbiota, relevant to the mucosal immunity and host metabolism, is likely the most crucial environmental factors in IBD pathogenesis. Multiple studies have confirmed that gut microbiota plays an key role in the severity and progression of colitis in IBD patients (Marion-Letellier et al., 2016). Although there is a strong linkage between the gut microbiota and IBDs, the exact mechanisms of the interactions are still unclear (Von Schillde et al., 2012).

Dextran sulfate sodium (DSS) administration is a chemical method to induce colitis, which results in similar key clinical and pathological features of IBDs to those in humans (Okayasu et al., 1990). DSS administration is believed to impair intestinal epithelial cells due to its toxicity. Consequently, the destroyed barrier function allows the immune cells to be exposed to antigens (e.g., microbes), resulting in a subsequent immune response (Wirtz et al., 2017). Based on the properties of colitis, this method is suitable to investigate the impacts of the intestinal microbiota on colitis progression (Hudcovic et al., 2001; Hernandez-Chirlaque et al., 2016). For instance, a recent study revealed that some specific bacterial taxa were present in the crypts due to the functions of NOD-like receptor protein 6 (NLRP6), which are associated with the severity of colitis (Elinav et al., 2011). Moreover, DSS-induced colitis has been broadly applied as a model for its simplicity and reproducibility.

*Akkermansia muciniphila* has been identified as the representative of the phylum *Verrucomicrobia* and represents 1–5% of the human intestinal microbial community (Collado et al., 2007; Derrien et al., 2008). *A. muciniphila*, as a gram-negative and strictly anaerobic bacterium, tends to widely colonize the nutrient-rich intestinal mucus layer (Derrien et al., 2004). Studies have demonstrated that *A. muciniphila* is able to degrade host mucin into various products (e.g., short chain fatty acids) to regulate host biological functions, such as glucose and lipid metabolism, and to modulate the expression of mucus-related genes, which participate in host immune responses (Derrien et al., 2004, 2011).

*A. muciniphila* was confirmed to exert a major role in maintaining gut barrier function, host metabolism and other biological functions through interactions between intestinal microbes and host in metabolic disorders (Everard et al., 2013). Moreover, Png et al. (2010) observed that the abundance of *A. muciniphila* was markedly reduced in patients with IBD compared with the abundance in controls, indicating that *A. muciniphila* may be related to the health of the intestinal mucosa. These findings indicate that *A. muciniphila* might play a crucial role in protecting against intestinal injury, which occurs in IBD. However, the precise protective roles *A. muciniphila* plays and the underlying mechanisms during the progression of the disease are not completely understood. Therefore, our study aimed to investigate the protective effects of *A. muciniphila* against colitis and the potential underlying mechanisms in a DSS-induced colitis mouse model.

## Materials and Methods

### Bacterial Strains and Growth Conditions

*A. muciniphila* Muc^T^ (ATTC BAA835) was cultured in a basal mucin-based brain heart Infusion medium (OXOID, Thermo Fisher Biochemicals Ltd., Baesingstoke, United Kingdom) at 37°C for 48 h under an anaerobic environment, as previously reported (Derrien et al., 2004). The cultures were centrifuged (8000 × g for 10 min at 4°C), washed with sterile phosphate buffer saline (PBS, pH 7.2) twice, and then concentrated to the final concentration of 1.5^∗^10^10^ colony-forming units (CFU)/ml by measuring absorbance at 630 nm (O.D. range from 0.6 to 0.8) for further use. The experiments described above were performed under strict anaerobic conditions.

### Animals

All animal experiments were reviewed and approved by the Local Committee of Animal Use and Protection. Specific pathogen-free (SPF) male C57BL/6 mice aged 5 weeks were initially obtained from Shanghai Laboratory Animal, Co., Ltd. (SLAC). All mice were conventionally raised under SPF conditions, and the experiments were performed when the mice were 6–7 weeks old.

### DSS-Induced Colitis and Experimental Design

**FIGURE 1:**
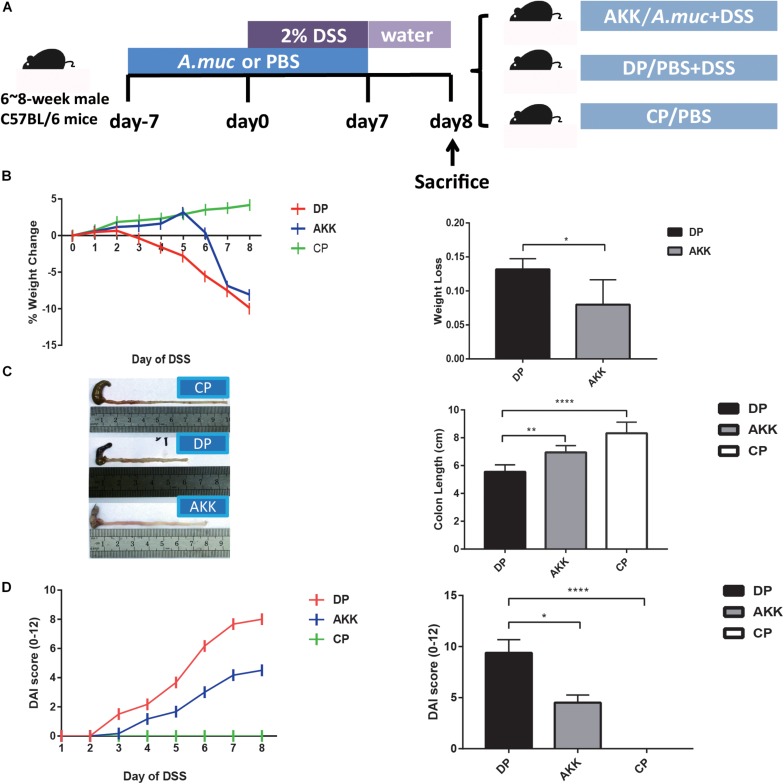
*Akkermansia muciniphila* alleviated DSS-induced colon injury **(A)** Scheme of the animal experimental design. Mice were assigned to three groups randomly (Group CP, *n* = 8; Group DP, *n* = 8; Group AKK, *n* = 8). Mice were continuously administered *A. muciniphila* or PBS for 14 days (day -7 to day 7). Colitis was induced by drinking 2% DSS from days 0 to 6, followed by 2 days (days 7–8) of normal drinking water. On day 8, mice were sacrificed. **(B)** The percentage of mouse weight change from days 0 to 8 (left) and the total weight loss relative to the baseline (day 0) (right). **(C)** Representative colons of three groups (left) and the colon length after DSS administration (right). **(D)** The disease activity index (DAI) scores calculated with time (left) and the bar chart represents the DAI scores among the groups at day 8 (right). Data are shown as the mean ± SEM. ^∗^*P* < 0.05; ^∗∗^*P* < 0.01; ^****^P < 0.0001 by unpaired *t*-tests, Kruskal–Wallis tests and *post hoc* one-way ANOVA.

Twenty-four mice were randomly assigned to three groups (*n* = 8 each): normal control group (CP group), experimental model group (DP group) and *A. muciniphila* group (AKK group). Mice in the AKK group (DSS + *A. muciniphila*) were administered 0.2 ml *A. muciniphila* (3^∗^10^9^ CFU) once daily by oral gavage for 14 days (day -7 to day 7, Figure 1A), while mice in the DP group (DSS + PBS) and the CP group (Water + PBS) were given equal amounts of anaerobic sterile PBS. On day 0, the mice in the AKK group and the DP group were administered 2% (wt/vol) DSS (molecular weight: 36,000–50,000; MP Biomedicals, cat. no. 0216011080) dissolved in drinking water for 7 days (days 0–6) followed by 2 days of normal water. New fresh DSS solutions were prepared every 2nd day. Meanwhile, the mice in the CP group were given normal drinking water for 9 days (days 0–8). Mouse weights were recorded throughout the experiment and fecal samples were collected on days 0 and 8. At the end of the experiment (day 8), all mice were humanely euthanized after serum and colon segment collection (Figure 1A). Feces and serum were stored at −80°C until analysis. Colon segments were either kept in 10% neutral-buffered formalin or stored at −80°C for further procedures.

### Disease Activity Index (DAI) Assessment and Severity of Colitis

The severity of colitis was measured using DAI assessment, a scoring system which includes three parts: body weight loss (0–4), degree of intestinal bleeding (0–4) and stool consistency (0–4) (Wirtz et al., 2017). The colon length, another indicator of colitis severity, was also measured when the mice were sacrificed.

### Histopathological and Immunofluorescence Analysis

Distal colon segments in the same defined region were collected carefully and were transferred immediately to 10% neutral-buffered formalin. After paraffin embedding, colon fragments were generated into 5 μm cross-sections. The sections were stained with hematoxylin and eosin (H&E) and then scanned by a NanoZoomer Digital Pathology system (Hamamatsu Photonics, KK, Japan) for further histopathological analysis. At least three slides were randomly selected and observed by a blinded pathologist. The histological activity index (HAI) is an established scoring system based on inflammatory cell infiltration (1–3), both in terms of severity and extent, and intestinal architecture (1–3), which includes epithelial changes and mucosal architecture, as previously described (Erben et al., 2014).

The colon sections were stained with the intestinal barrier markers *ZO-1* and *Occludin* (both Proteintech, Rosemont, IL, United States) with a previously described standard immunofluorescence analysis process (Chung et al., 2014). Images were captured and visualized using a Zeiss LSM T-PMT confocal microscope (Zeiss, Jena, Germany).

### Intestinal Permeability

Intestinal permeability was determined by the fluorescein isothiocyanate conjugated dextran (FITC-dextran) assay. Mice were fasted overnight and were administered FITC-dextran (3–5 kDA, Sigma-Aldrich, 60 mg/100 g body weight) by oral gavage 4 h before blood collection. The concentration of FITC was determined by spectrophoto fluorometry (490/525 nm).

### Serum Parameter Analysis

Lipopolysaccharide (LPS)-binding protein (LBP) concentrations, an indicator of endotoxin levels, were measured using an appropriate ELISA kit (Guduo, Shanghai, China) as previously reported (Ye et al., 2018).

A Bio-Plex Pro mouse cytokine 23-plex assay kit (Bio-Rad, Hercules, CA, United States) were performed to analyze the serum cytokine levels according to the manufacturer’s instructions. The 23-plex contains the following antibodies specific for cytokines and chemokines: interleukin (IL)-1α,IL-1β, IL-2, IL-3, IL-4, IL-5, IL-6, IL-9, IL-10, IL-12 (p40), IL-12 (p70), IL-13, IL-17, Interferon (IFN)-γ, tumor necrosis factor alpha (TNF-α), eotaxin, granulocyte colony-stimulating factor (G-CSF), granulocyte-macrophage-colony-stimulating factor (GM-CSF), keratinocyte-derived chemokine (KC), macrophage chemoattractant protein 1 (MCP-1), macrophage inflammatory protein (MIP)-1α, MIP-1β and regulated upon activation, normal T-cell expressed and secreted (RANTES).

### Determination of Colonic mRNA Expression

An RNeasy Plus Mini Kit (Qiagen, Valencia, CA, United States) was used to extract total colon tissue RNA according to the manufacturer’s procedures. The reverse transcription for two-step reverse transcription polymerase chain reaction (RT-PCR) was performed using a PrimeScript RT Master Mix reverse transcription reagent kit (Takara Biomedicals, Kusatsu, Japan). The relative mRNA expression was measured and analyzed by a ViiA7 real-time PCR system (Applied Biosystems) using a SYBR Premix Ex Taq II (Tli RNase H Plus) reagent (Takara Biomedicals, Kusatsu, Japan). All samples were tested in duplicate and assessed using the 2^–ΔΔ*CT*^ method before the results were normalized to the expression of β-actin. The final results of the AKK and DP groups were calculated relative to the CP group. Primer pairs used are presented in Supplementary Table S1.

### Fecal Microbial Community Analysis

Fecal samples were immediately frozen and stored at −80°C freezer after collection. Total bacterial DNA was extracted from fecal samples (approximately 200 mg) using a QIAamp Fast DNA Stool Mini Kit (Qiagen, Hilden, Germany) following the manufacturer’s procedures. The V3–V4 regions of the bacterial 16S rRNA were amplified by Phusion High-Fidelity PCR Master Mix (New England Biolabs) using the specific primer (e.g., 16S V4:515F-806R) with a barcode. After PCR product purification, sequencing libraries, were generated using Ion Plus Fragment Library Kit 48 rxns (Thermo Scientific) following the manufacturer’s instructions, The library quality was assessed on the Qubit@2.0 Fluorometer (Thermo Scientific). At last, the library was sequenced was on an Ion S5^TM^ XL platform (Thermo Scientific) and 400 bp/600 bp single-end reads were generated. Clean reads were finally obtained after single-end read quality controlling procedures. Sequences analyses were performed by Uparse software (Uparse v7.0.1001) (Edgar, 2013). Sequences with ≥97% similarity were assigned to the same OTUs. Representative sequence for each OUT was screened for further annotation.

Taxonomic information was annotated based on the Silva Database^1^ for each representative sequence. Alpha diversity indices, including Chao1, ACE, Shannon and Simpson, were calculated with QIIME (Version 1.7.0). Principal coordinate analysis (PCoA) was performed by R software (version 2.15.3) based on weighted UniFrac distances. Linear discriminant analysis (Yatsunenko et al., 2012) effect size measurement (LefSe) analysis was conducted online (Bajaj et al., 2014). Spearman’s rank correlation test was performed to analyze the relationships between the microbiota and colitis-related indices in AKK group and DP group using SPSS version 20.0 (SPSS, Inc., Chicago, IL, United States), and Cytoscape was used to visualize the results graphically. Prediction of metabolic functions of the microbiota was performed by using Phylogenetic Investigation of Communities by Reconstruction of Unobserved States (PICRUSt V1.0.0).

### Short Chain Fatty Acid Quantification

Short chain fatty acids (SCFAs) were extracted by mixing 20 mg of feces with 500 μl internal standard (hexanoic acid-d3, 10 μg/ml). After homogenization and centrifugation (15,000 rpm, 5 min, 4°C), the supernatant was transferred into an Eppendorf tube with 5% concentrated sulfuric acid (supernatant vs. 5% concentrated sulfuric acid, volume 10:1). Then, an equivalent volume of ethyl acetate was added to the mixture. After centrifugation and incubation at 4°C for 30 min, the supernatant was transferred to chromatographic vials equipped with 150 μl inserts before the gas chromatography mass spectrometry (GC-MS) analysis. The analysis was performed on an Agilent 7890A GC oven coupled to an Agilent 5975C inert mass selective (MS) detector (Agilent Technologies, Santa Clara, CA, United States). SCFAs were identified by the times of retention in accordance with the standard solutions. Standard mixes of 0.01, 0.1, 1, 10, 100, 1000 μl/ml and blank were also prepared in the same way as the samples.

### Statistical Analysis

The Kolmogorov-Smirnov test was performed to assess the normality of the data. Unpaired *t* tests with Welch’s correction were used to determine weight loss difference between the DP and AKK groups. Colon length, serum cytokines, colonic cytokine expression, *LBP*, Shannon index, relative abundance, FITC-dextran and SCFAs were analyzed by *post hoc* one-way ANOVA. Kruskal–Wallis tests were performed to analyze the DAI and histopathology score among groups. A multi response permutation procedure (MRPP) test was used to analyze the β diversity in fecal analysis. Correlations analysis was conducted using Spearman’s rank correlation. *P* < 0.05 were considered significant. All data analyses were performed using SPSS version 20.0 (SPSS, Inc., Chicago, IL, United States) and GraphPad Prism 7.0 (GraphPad Software, Inc.), with a two-tailed *P* < 0.05 considered significant, and the results are presented as the mean ± SEM.

## Results

### *A. muciniphila* Alleviated DSS-Induced Colitis

As reported previously, body weight loss during DSS model establishment can be used as a metric of colitis severity because it is easily measured and is consistent with the inflammatory markers (Llewellyn et al., 2018). The percentage of weight change was measured daily for 9 days relative to their baseline body weight that was recorded on day 0. As Figure 1B shows, the body weights of the mice in the DP group were consistently reduced since day 2, while the mice in the AKK group mice showed a significant improvement in weight loss, although there was an apparent decrease on day 6. At the end of the experiment, reduced body weight loss was observed in the mice in the AKK group compared to the mice in the DP group (8.00 ± 1.49% vs. 13.17 ± 0.64%, *P* < 0.05). In the colitis model, disease severity is typically associated with colon length shortening due to intestinal inflammation (Wirtz et al., 2017). Following the 7 days of DSS exposure, the colons of the mice were shorter in the DP group than in the CP group (*p* < 0.0001) and the AKK group (p < 0.01) (Figure 1C). Importantly, administration of DSS induced weight loss and bloody diarrhea in the mice, and these factors are inflammation-associated parameters resembling some clinical features of human UC and can be used to calculate the DAI score. In our research, the score increased with the time of DSS administration and was much higher in the mice in the DP group than in those in the CP group (*P* < 0.0001) and the AKK group at the end of the experiment (*P* < 0.05) (Figure 1D).

**FIGURE 2:**
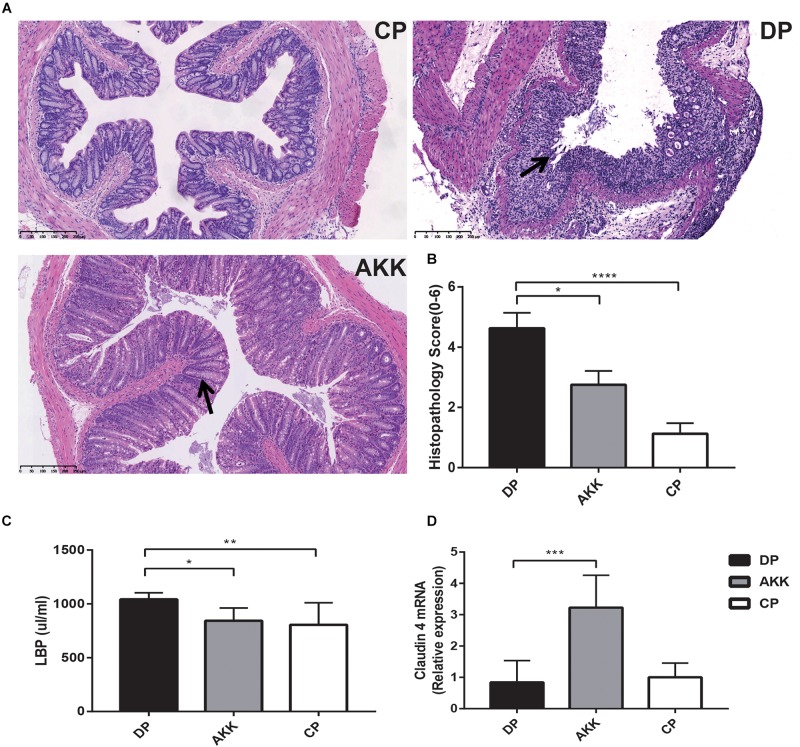
Akkermansia muciniphila ameliorated colon mucosal barrier damage in mice **(A)** Representative H&E staining of colon tissues of three groups. The black arrows show epithelial damage (scale bar: 100 μm). **(B)** Histopathology scores (0–6) of colon tissues and colon expression genes of **(C)** *LBP* and **(D)** *claudin 4*. Data are shown as the mean ± SEM. ^∗^*P* < 0.05; ^∗∗^*P* < 0.01; ^∗∗∗^*P* < 0.001; ^****^P < 0.0001 by Kruskal–Wallis tests and *post hoc* one-way ANOVA.

As far as we know, all chemically induced colitis models, including the oral administration of DSS, the method we used in our experiment, begin with epithelial barrier disruption. H&E colon staining (Figure 2A) showed that DSS-induced colitis resulted in extended ulcerations, destroyed crypts and transmural inflammatory infiltration, with a barely complete mucosal structure. However, in mice treated with *A. muciniphila*, histological damage was ameliorated, as evidenced by a preserved mucosal architecture showing focal erosions and mild/moderate mucosa inflammatory infiltration. To measurably evaluate the intestinal mucosal architecture (0–3) and inflammatory infiltration (0–3), we used the histopathology score (0–6) system (Erben et al., 2014), and the mice treated with *A. muciniphila* had a significantly lower score than the mice treated with DSS only (*P* < 0.05) (Figure 2B).

### *A. muciniphila* Ameliorated Colon Mucosal Barrier Damage in Mice

Colon epithelial destruction and inflammatory activity increase the risk of intestinal bacterial translocation, which in turn aggravates colitis severity (Lakatos et al., 2011). Therefore, serum LBP was measured by ELISAs to assess bacterial translocation and colitis activity. The results (Figure 2C) revealed that serum LBP levels in the DP group were higher than the levels in the CP and AKK groups (*P* < 0.01, *P* < 0.05, respectively).

**FIGURE 3:**
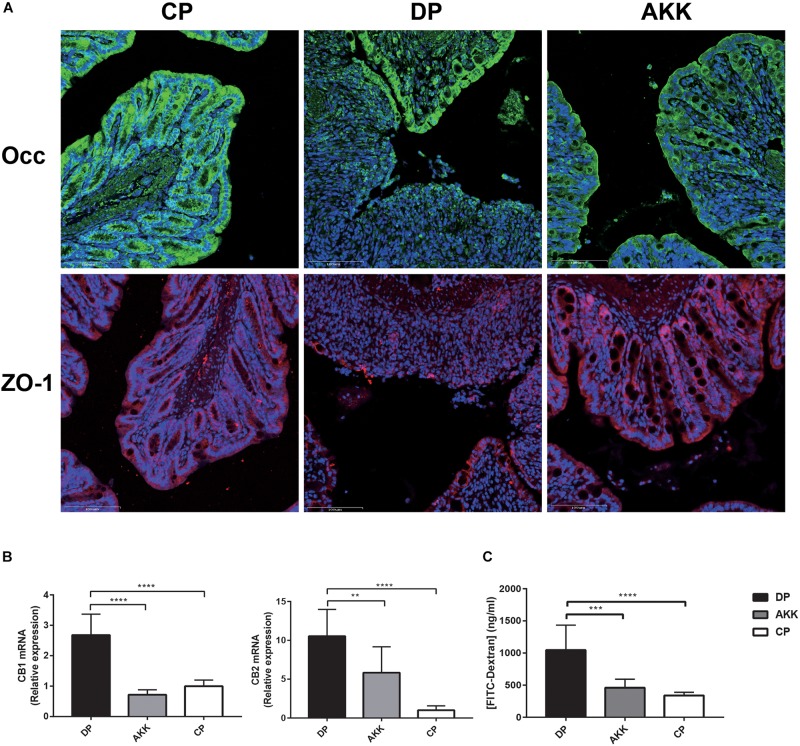
*Akkermansia muciniphila* enhanced gut barrier function **(A)** *Occludin* and *ZO-1* immunofluorescence staining of representative colon tissue (scale bar: 100 μm). **(B)** Colon expression of genes related to gut barrier function, including *CB1* and *CB2*. **(C)** Intestinal permeability measured by determining the plasma concentration of FITC-dextran. Data are shown as the mean ± SEM. ^∗∗^*P* < 0.01; ^∗∗∗^*P* < 0.001; ^****^P < 0.0001 by *post hoc* one-way ANOVA.

Intestinal mucosal barrier destruction was associated with colitis progression; additionally, junction protein expression (*occludin*, *ZO-1*, and *claudin-4*) in the colon was determined by quantitative PCR and immunofluorescence. *Claudin-4* mRNA expression was decreased in the colon tissue of the DP group; in contrast, the AKK group had an upregulated level (*P* < 0.001, Figure 2D). Notably, *occludin* mRNA expression in the colon was downregulated in the DP group compared with that in the CP group (*P* < 0.001). However, no significant difference in *occludin* and *ZO-1* mRNA expression was found between the AKK and DP groups (data not shown). As Figure 3A shows, the colon tissue in the DP group presented increased TJ structure defects with destroyed crypts and a disrupted apical region, while *A. muciniphila* administration stabilized mucosal integrity, resulting in smooth and continuous localization of *occludin* and *ZO-1*.

Furthermore, cannabinoid receptor (CB) 1 and CB2 were positively associated with maintaining gut permeability and plasma LPS levels (Muccioli et al., 2010). Consistent with the serum LBP level, the expression of these two markers (CB1 and CB2) was significantly increased after DSS administration (*P* < 0.0001, *p* < 0.0001), while the expression was downregulated in mice treated with *A. muciniphila* (*P* < 0.0001, *P* < 0.01, Figure 3B). Notably, FITC-dextran analysis showed a decreased intestinal permeability in mice of the AKK group compared to those of the DP group, suggesting an increased barrier function following *A. muciniphila* treatment (*P* < 0.001, Figure 3C).

### *A. muciniphila* Treatment Resulted in Systemic and Intestinal Anti-inflammation

**FIGURE 4:**
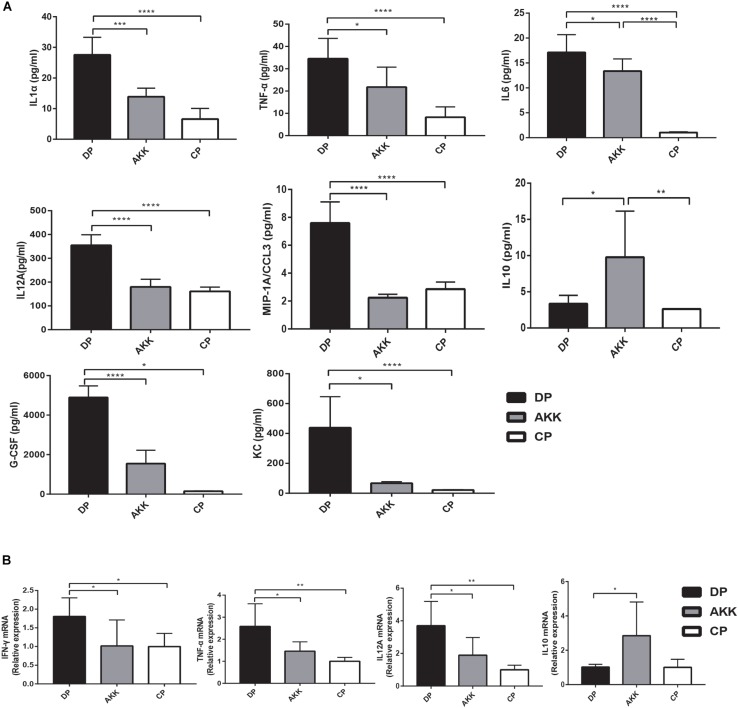
*Akkermansia muciniphila* exhibited systemic and intestinal anti-inflammatory effects. **(A)** Bar charts represent the serum cytokine levels of *IL-1*α*, TNF-*α*, IL6, IL12A, MIP-1A*, and *IL10* among the groups. **(B)** Colonic cytokine expression of *TNF-*α*, IL12A, IFN*γ, and *IL10*. Data are shown as the mean ± SEM. ^∗^*P* < 0.05; ^∗∗^*P* < 0.01; ^∗∗∗^*P* < 0.001 by *post hoc* one-way ANOVA.

Colon inflammatory cell infiltration increases mucosal production of cytokines. Pro-inflammatory cytokines play an crucial role in the progression of DSS-induced colitis (Andlujar et al., 2011). To explore whether *A. muciniphila* treatment could alleviate colitis by regulating inflammation, we detected the serum cytokines and cytokine expression in distal colon tissue. An increase in serum cytokines, including the pro-inflammatory cytokines TNF-α, IL1α, IL6, and IL12A, as well as the chemokines MIP-1A, G-CSF, and KC, was observed in mice administered DSS only (DP vs. CP, Figure 4A). In the AKK group, these cytokines were significantly downregulated (*P* < 0.05, *P* < 0.001, *P* < 0.05, *P* < 0.0001, *P* < 0.0001, *P* < 0.0001, *P* < 0.05, respectively), and their levels were similar to those in the CP group. In addition, similar results were found in colon tissue inflammatory expression (Figure 4B). The expression levels of TNF-α (*P* < 0.01), IL12A (*P* < 0.01), and INFγ (*P* < 0.05) were significantly upregulated in the DP group compared to those observed in the CP group. In contrast, TNF-α (*P* < 0.05), IL12A (*P* < 0.05), and INFγ (*P* < 0.05) were downregulated compared with the levels in the DP group and were almost reduced to the normal level after administration of *A. muciniphila*. IL1α, IL6, and MIP-1A showed similar decreasing trends (data not shown). Furthermore, in mice treated with *A. muciniphila*, the immunomodulatory cytokine IL10 expression level was increased both in the serum (*P* < 0.05, Figure 3A) and colon tissue (*P* < 0.05, Figure 3B). The remaining serum cytokines tested are shown in the Supplementary Figure S1.

### *A. muciniphila* Alleviated DSS-Induced Microbiome Dysbiosis and Reshaped the Gut Microbiota Community

**FIGURE 5:**
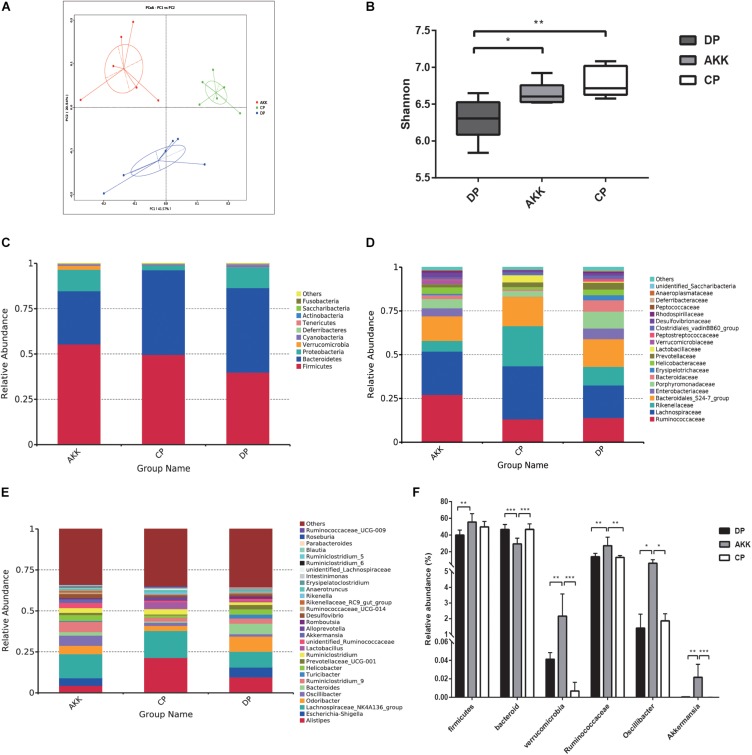
*A. muciniphila* alleviated DSS-induced microbiome dysbiosis. **(A)** PCoA analysis among three groups based on weighted UniFrac distances. Each plot represents one sample. **(B)** The Shannon index among three groups. **(C–E)** Relative abundance of taxa at the phylum **(C)**, family **(D)** and genus **(E)** levels. **(F)** Relative abundance of taxa in the DP group and AKK group. Data are shown as the mean ± SEM. ^∗^*P* < 0.05; ^∗∗^*P* < 0.01; ^∗∗∗^*P* < 0.001; ^****^P < 0.0001 by *post hoc* one-way ANOVA.

Gut microbes are an important factor in intestinal inflammation (Tamboli et al., 2004; Sartor and Wu, 2017). To further explore the protective impact of *A. muciniphila* supplementation on mice with DSS-induced colitis, 16S rRNA sequencing analysis was conducted to assess alterations in the gut microbiota. The sequencing data have been deposited in the sequence read archive (SRA) database with the accession PRJNA 54980. Fecal samples were collected from three groups (CP group = 6, DP group = 6, AKK group = 6) after (day 8) DSS administration. Rarefaction curves of three groups reflected the sequencing depth of the fecal analysis (Supplementary Figure S2). A total of 1,285,692 clean reads (AKK = 431,984, CP = 427,244, DP = 426,464) were obtained for further analysis and 11,982 identified OTUs (AKK = 3,853, CP = 4,530, DP = 3,599) were clustered with a 97% similarity cutoff. In contrast to the CP group, the fecal microbiome of the mice administered DSS (DP group) exhibited reduced α-diversity, which was reflected in the decrease in observed species (*P* < 0.01) and the Ace (*P* < 0.001), Chao1 (*P* < 0.01), and Shannon (*P* < 0.01) indices (data not shown). The Shannon index, an estimator used to identify community diversity, was observed to be significantly higher in mice treated with *A. muciniphila* than those not treated with *A. muciniphila* (*P* < 0.05, AKK vs. DP, Figure 5B). Next, we performed weighted UniFrac PCoA analysis to investigate the differences in species complexity and structural alterations in gut microbial communities (β diversity). The results revealed significant different β diversity among three groups (Figure 5A and Supplementary Table S2). The DP group samples were separated from the CP group samples (*P* = 0.002, Supplementary Table S2). Likewise, the microbiota of the AKK and DP group samples were clearly separated into two clusters (*P* = 0.002, Supplementary Table S2), demonstrating that continuous administration of *A. muciniphila* alleviated DSS-induced dysbiosis of the gut microbiome.

The relative abundance of bacteria taxa was altered in individuals with IBD (Zuo and Ng, 2018). Therefore, we used Wilcoxon rank sum tests to compare the relative taxa abundance between the three groups. The top most abundant taxa at the phylum, family and genus levels are shown in Figures 5C–E. Previous studies have clarified that *Firmicutes* and *Bacteroidetes* are two dominant phyla in mouse fecal samples (Wu et al., 2017). At the phylum level (Figure 5C), compared to the CP group, the abundance of *Firmicutes* was decreased and that of *Bacteroidetes* was increased as a result of DSS oral administration (F/B < 1). In contrast, there was a marked increase in the proportion of *Firmicutes* (*P* < 0.01, vs. DP) and a decrease in *Bacteroidetes* (*P* < 0.001 vs. DP, *P* < 0.001 vs. CP) after continuous administration of *A. muciniphila*. Additionally, the abundance of *Verrucomicrobia* was also increased due to the treatment (*P* < 0.001 vs. DP, *P* < 0.001 vs. CP). At the family level, the relative abundance of *Ruminococcaceae* (*P* < 0.01) in the AKK group was increased compared with that in the DP group (Figure 5D), while the relative abundances of *Rikenellaceae* and *Enterobacteriaceae* were decreased, although there was no significant difference. At the genus level, the fecal microbiota of the AKK group had a significantly higher abundance of *Oscillibacter* than that of the DP group (*P* < 0.05) and CP group (*P* < 0.05, Figure 5F).

**FIGURE 6:**
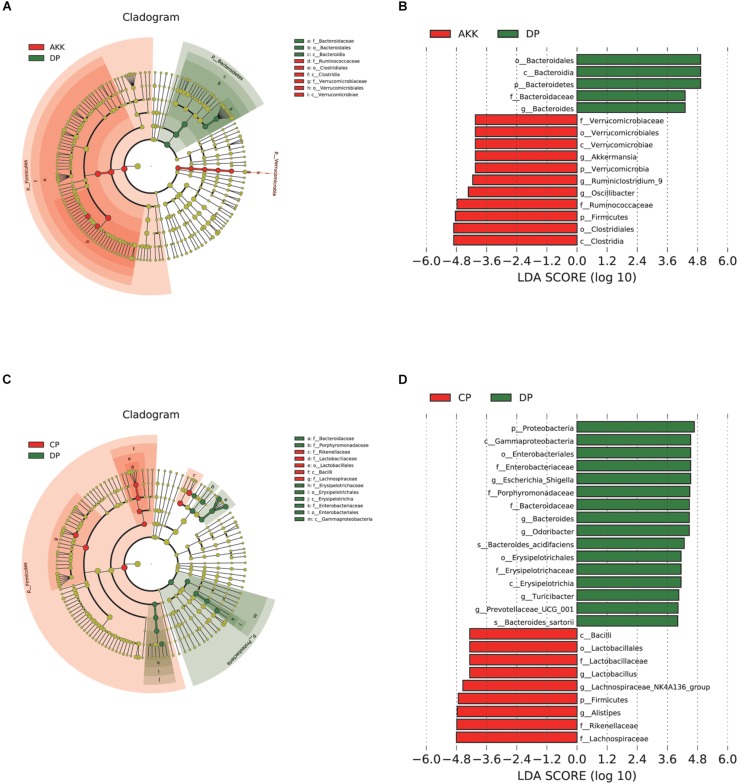
*Akkermansia muciniphila* reshaped the gut microbiota community. **(A)** LEfSe cladogram represents the taxa enriched in the AKK group (Red) and DP group (Green). **(B)** Discriminative biomarkers with an LDA score >4.8 between the AKK group (Red) and DP group (Green). **(C)** LEfSe cladogram represents taxa enriched in the CP group and DP group. **(D)** Discriminative biomarkers with an LDA score >4.8 between the CP group (Red) and DP group (Green).

Studies have shown that microbial markers might be instruments that can be used for personalized therapy (Zuo and Ng, 2018). Therefore, we performed LEfSe analysis to explore the prognostic biomarkers from the microbial abundance profiling between the three groups. We found that treatment with *A. muciniphila* increased the abundance of *Clostridia*, *Firmicutes*, *Ruminococcaceae*, *Oscillibacter*, *Verrucomicrobia*, and *Akkermansia* [LDA score (−log10) > 4.8] and decreased the abundance of *Bacteroidetes* [LDA score (log10) > 4.8] (Figures 6A,B). Interestingly, due to DSS administration, the fecal flora *Enterobacteriales*, *Escherichia Shigella*, and *Erysipelotrichales* were enriched, and there was a depletion of *Lactobacillales*, *Rikenellaceae, and Lachnospiraceae* (Figures 6C,D). Regarding the Kyoto *Encyclopedia* of Genes and Genomes (KEGG) metabolic pathways, several predicted functional differences were observed between the DP and AKK groups, such as the NOD-like receptor signaling pathway, the glycosaminoglycan degradation pathway, and the lipid metabolism pathway (Supplementary Figure S3).

**FIGURE 7:**
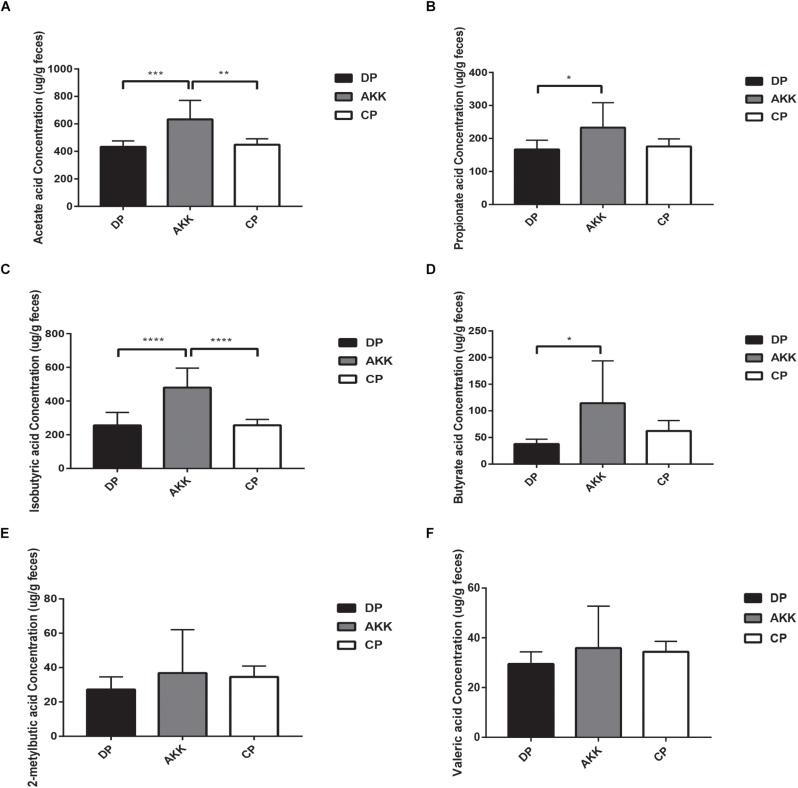
Short-chain fatty acids (SCFAs) distribution observed in three groups. The fecal concentrations of acetate acid **(A)**, propionate acid **(B)**, isobutyric acid **(C)**, butyrate acid **(D)**, 2-metylbutic acid **(E)**, and valeric acid **(F)** were determined by GC–MS. Data are shown as the mean ± SEM. ^∗^*P* < 0.05; ^∗∗^*P* < 0.01; ^∗∗∗^*P* < 0.001; ^****^P < 0.0001 by *post hoc* one-way ANOVA.

A total of six SCFAs, including acetate acid, propionate acid, isobutyric acid, butyrate acid, 2-metylbutic acid and valeric acid were identified in our study from 24 fecal samples in all three groups. The acetate acid, propionate acid, isobutyric acid and butyrate acid levels were significantly increased in the AKK group (vs. DP group, *P* < 0.001, *P* < 0.05, *P* < 0.0001, and *P* < 0.05, respectively, Figures 7A–D). However, there was no change observed in 2-metylbutic acid and valeric acid levels with *A. muciniphila* treatment (Figure 7).

### Correlation Analysis Indicated That the *A. muciniphila*-Modified Gut Microbiota Played an Essential Role in the Progression of DSS-Induced Colitis

**FIGURE 8:**
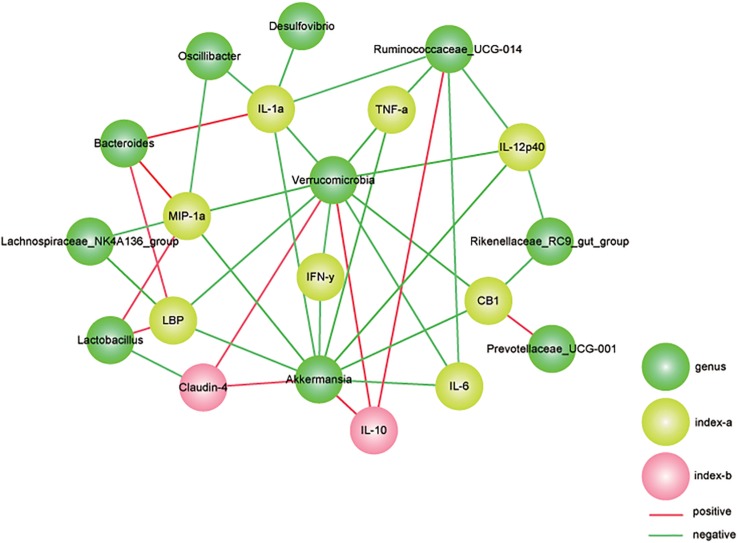
Correlation analysis between gut microbiota and colitis parameters. We used Spearman’s rank correlation and significant relationships with *P* < 0.05 and *r* > 0.5 are displayed. Yellow nodes represent the colitis parameters. Green nodes represent the differentially distributed genus. Green lines represent negative relationships, and the red lines represent positive relationships. The thickness of the line indicates the value of r.

To verify the relevance of the altered microbial community and the severity of colitis, a correlation analysis between gut bacteria abundance and colitis parameters was performed. In our study, the relative abundances of *A. muciniphila*-modified bacteria were associated with gut barrier markers and related inflammatory cytokines (Figure 8). As one of the representative colitis severity markers, TNF-α was negatively related to *Verrucomicrobia, Ruminococcaceae_UCG-014*, and *Akkermansia.* Additionally, the primary pro-inflammatory cytokine IL12 (p40) was negatively associated with the population densities of *Ruminococcaceae_UCG-014, Verrucomicrobia*, *Akkermansia*, and *Rikenellaceae_RC9_gut_group.* Notably, we found that *Verrucomicrobia*, was prominently abundant in the AKK group and that it was significantly negatively correlated with all tested gut barrier markers (claudin-4, LBP, and CB1) and inflammatory cytokines, including TNF-α,IFN-γ, IL12 (p40), IL1α, and IL6. In contrast, *Bacteroides*, which were enriched in the DP group, were positively associated with MIP-1α, IL1α, and LBP. The anti-inflammatory cytokine IL10, upregulated due to *A. muciniphila* administration as mentioned above, was markedly positively correlated with *Verrucomicrobia, Ruminococcaceae_UCG-014*, and *Akkermansia.*

## Discussion

Recently, several studies have demonstrated that the newly isolated bacterium *A. muciniphila Muc*^*T*^ is highly associated with healthy mucosa in the context of multiple disorders (Derrien et al., 2011; Shin et al., 2014; Plovier et al., 2017). A similar finding was observed in IBD patients, suggesting that *A. muciniphila* may exert a beneficial effect during the progression of colitis (Png et al., 2010). However, the protective effects of *A. muciniphila* have not been widely demonstrated and its microbial and immunity moderation properties in colitis. Therefore, we determined the impacts of *A. muciniphila* using a DSS-induced colitis mouse model. In our study, we found that *A. muciniphila* could substantially ameliorate the colitis, as indicated by lower body weight loss, colon length shortening, histological scores and DAI scores. Additionally, some of the potential mechanisms were presented, including (i) amelioration of colon mucosal barrier damage; (ii) modulation of the inflammatory response; (iii) reshaping the gut microbiota; and (iv) regulation of metabolic functions.

As previously reported, thinner colonic mucus was found in patients with UC, as well as in mouse and rat models of colitis, compared with controls (Petersson et al., 2011; Dicksved et al., 2012; Johansson et al., 2014). Furthermore, multiple studies have revealed that intestinal permeability and LPS levels were increased in mice with colitis (Souza et al., 2015; Llewellyn et al., 2018), these findings suggested intestinal barrier damage and sequentially aggravated intestinal colitis. Importantly, *A. muciniphila*, identified as a mucin-degrading bacteria (Derrien et al., 2004), could continuously reshape and refresh the mucus layer, thereby creating a healthy environment for epithelial cells and maintaining the integrity of the intestinal barrier (Everard et al., 2013). *In vitro*, Reunanen et al. (2015) found that *A. muciniphila* could strengthen Intestinal epithelial integrity and fortify a destroyed gut barrier. Our findings of decreased serum LBP levels, strengthen tight junctions, and reduced CB1 and CB2 expression due to *A. muciniphila* administration were in agreement with the results of previous studies (Lin et al., 2012).

Accumulating evidence has revealed that the gut microbiota regulates the gut endocannabinoid (eCB) system, which in turn controls intestinal barrier function and inflammation by modulating intestinal permeability and LPS levels, but the molecular link between the colonic eCB system and IBD remains unknown (Maccarrone et al., 2001; Di Marzo et al., 2009; Hoareau et al., 2009). Muccioli et al. (2010) also found that there was a close relationship between the intestinal eCB system and LPS and that LPS levels were reduced by CB1 blockage through a mechanism related to the integrity of the intestinal barrier. Crucially, *A. muciniphila* treatment was observed to increase the colonic levels of endocannabinoids (Everard et al., 2013). In the current study, the expression of CB1 was upregulated after DSS administration, and *A. muciniphila* supplementation markedly downregulated the CB1 level, indicating an important role of *A. muciniphila* in improving gastrointestinal function through eCB function.

Importantly, the serum and colon tissue inflammatory cytokine levels in the AKK group showed significant inflammation relief, suggesting that *A. muciniphila* may have an important impact on systemic and intestinal anti-inflammation in mice with DSS-induced colitis.

Epithelium damage and profound intestinal inflammation were observed in IBD patients (Souza et al., 2005; Coskun, 2014). Likewise, during DSS administration, the colonic immune cells were exposed to luminal antigens as a result of the injured intestinal epithelium, subsequently inducing a strong inflammatory immune response (Wirtz et al., 2017). Our results demonstrated that *A. muciniphila* treatment exerted a protective effect against mouse colitis by inhibiting some critical components associated with the inflammatory response, including pro-inflammatory cytokines (TNF-α, IL6, IL12A, INF-γ, and IL1α), anti-inflammatory cytokines (IL-10), and chemokines (MIP-1α, G-CSF, and KC). A previous study also observed that the expression of genes associated with the immune response was increased by *A. muciniphila* colonization (Derrien et al., 2011). Cytokines such as TNF-α and IL6 were highly expressed in humans with IBD (Reinecker et al., 1993) and experimental models of IBD (Yoshida et al., 2011; Senchenkova et al., 2013; Yan et al., 2014), and interventions to block their accumulation could protect against colitis (Souza et al., 2015). Moreover, these cytokines have been found to be involved in the disruption of epithelial tight junctions through the NF-κB signaling pathway, resulting in mucosal dysfunction (Fink, 2003; Ahl et al., 2016; Woodhouse et al., 2018; Li et al., 2019). In experimental colitis (Souza et al., 2015) and in IBD patients (Vainer et al., 1998; Banks et al., 2003), chemokines (e.g., MIP-1α) were produced at an accelerated rate and were involved in monocytes/macrophages activations and inflammatory immune modulation (Carr et al., 1994; Kostova et al., 2015). Our study consistently found that DSS administration markedly upregulated MIP-1α expression both in serum and colon tissue, and treatment with *A. muciniphila* could reverse the alteration.

IL-10, secreted by dendritic cells (DCs), monocytes, B cells, CD4^+^ T cells and CD8^+^ T cells, plays an important role in anti-inflammatory responses by binding the IL10R1 and IL10R2 receptors (Del Prete et al., 1993). Therefore, an IL10R intervention was commonly a way to study the correlation between the immune response and gut microbiota composition (Loren et al., 2017). Pre-treatment with *Lactobacillus* GG could prevent rat colitis associated with increased IL10 expression (Dieleman et al., 2003), and IL10 gene therapy could prevent TNBS-induced colitis in mice (Lindsay et al., 2002). *In vitro*, the anti-inflammatory function of IL10 has been demonstrated in a range of cell types (Ribbons et al., 1997), and increased IL10 subsequently reduces the underlying production of TNF-α and IL6 (Malefyt et al., 1991). Lammers et al. (2003) found that IL10 production was increased in human peripheral blood mononuclear cells (PBMCs) stimulated by components from certain VSL#3 bacteria. Importantly, IL10 exerts its preventative function before the development of inflammation, and it is ineffective once inflammation has occurred (Barbara et al., 2000; Lindsay et al., 2002; Van Montfrans et al., 2002). Consistent with the findings of these studies (Dieleman et al., 2003; Di Giacinto et al., 2005), our experiment indicated that the beneficial impact of *A. muciniphila* in experimental colitis was involved in the induction of IL10.

Compelling studies have revealed that broad intestinal microbial dysbiosis, including a reduction in bacterial abundance and a decrease in diversity, occurs in IBD (Manichanh et al., 2006; Frank et al., 2007; Dicksved et al., 2012; Putignani et al., 2016). Our results have consistently shown that there is an intestinal microbiota alteration pattern in mice administered DSS, revealing a decrease in α diversity and an altered relative abundance of specific bacteria taxa. In the group supplemented with *A. muciniphila*, we observed a restored α diversity and changes in fecal bacteria, including increased *Firmicutes* and decreased *Bacteroidetes*. However, restored β diversity in the group supplemented with *A. muciniphila* was not observed in our study, indicating that the *A. muciniphila* administration cannot fully reverse the gut microbiota construction in a state of colitis.

Specific bacteria in the *Enterobacteriacae* family were enriched both in mice and IBD patients (Lupp et al., 2007). In human studies, *Escherichia* was found in UC patients (Sokol et al., 2006) and ileac biopsies of individuals with CD (Darfeuille-Michaud et al., 2004). Similarly, the augmented *Escherichia/Shigella* levels may worsen disease severity by increasing intestinal permeability (Quigley et al., 2013). These data provide support for the conjecture that *Enterobacteriaceae* may have growth advantages in the inflammatory gut environment of IBD. Similar results were observed in our study in which *Enterobacteriacae* was clustered in mice treated with DSS, and overgrowth of this bacterial clade destroyed the intestinal permeability, resulting in worsened colitis.

A range of specific groups of microbes have the ability to protect the host from colitis. Some members of the gut microbiota were capable of modulating mucosal inflammation by stimulating IL10 secretion (Sokol et al., 2006). *Clostridium* species could protect the host from mucosal inflammation by recruiting regulatory T cells (Atarashi et al., 2013), and other gut bacterial groups can mitigate intestinal colitis via the NF-κB pathway (Kelly et al., 2004). The results obtained from our study consistently revealed that *Verrucomicrobiales* was correlated with a majority of tested factors involved in immune regulation. *Akkermansia* and *Ruminococcaceae* were positively associated with IL10 and negatively associated with IL6 and TNF-α. These microbes were significantly clustered in the group that was treated with *A. muciniphila*, indicating that their protective role was possibly modulated by the mucosal immune response.

Recent studies have revealed that *A. muciniphila* is able to degrade host mucin into various metabolites (e.g., SCFAs) to regulate host biological functions, such as glucose and lipid metabolism, and to modulate the expression of mucus-related genes associated with cell death receptor signaling, which participate in host immune responses (Derrien et al., 2004; Derrien et al., 2011). SCFAs could exert beneficial impacts on host energy metabolism (Zuo and Ng, 2018). SCFAs are fermented from dietary fiber by some gut microbes and are considered the primary energy source of gut epithelial cells (Ahmad et al., 2000). Several studies found that SCFAs have important immunomodulatory functions in innate immune cells (Fusunyan et al., 1999; Ciarlo et al., 2016). SCFAs, such as butyrate, can inhibit NF-κB activation and stimulate mucin production, resulting in an induced inflammatory response and enhanced barrier function (Mchardy et al., 2013). In addition, SCFAs are an important fuel for some gut commensals, which might increase the abundance of SCFA-dependent bacteria and modulate the interactions of gut commensals, resulting in the alteration of the microbiota composition and functions (Belzer and De Vos, 2012). Notably, the increased relative abundance of *Verrucomicrobia, Ruminococcaceae* and *Oscillibacter* in mice with *A. muciniphila*, which has not been observed in the CP and DP groups, might be due to *A. muciniphila* and its metabolites. *Firmicutes*, including the families *Lachnospiraceae* and *Ruminococcaceae*, is considered an anti-inflammatory factor due to their production of SCFAs, especially butyric acid. In humans, members of the *Lachnospiraceae* family may exert a protective effect against colon cancer (Meehan and Beiko, 2014), mainly due to the production of butyric acid, a substance that is associated with the gut microbiome, host epithelial cells and human physiology. Within these two families, some genera were positively correlated with anti-inflammatory cytokines and gut barrier markers and negatively correlated with pro-inflammatory cytokines, indicating protective impacts of these microbes on host health. These data implied that the favorable role of *A. muciniphila* in immune response was mediated not only by the strengthened gut barrier function, but also by the productions of beneficial bacteria.

To understand the functional interactions between microbes and hosts, we investigated the predicted functional differences in KEGG pathways. We observed that several predicted pathways, such as the NOD-like receptor signaling pathway, glycosaminoglycan degradation pathway, and the lipid metabolism pathway, differed between the DP group and the AKK group. Consistent with our results, Dunn et al. (2016) found that there was a significantly different NOD-like receptor signaling pathway between patients with CD and controls. The NOD-like receptor signaling pathway may contribute to host immune regulation (Shade and Handelsman, 2012; Tremaroli and Backhed, 2012; Guinane and Cotter, 2013; Larsen and Dai, 2015; Moore-Connors et al., 2016). Glycosaminoglycans (GAGs), including keratan sulfate and dermatan sulfate, are essential for the gut mucosa because they regulate gut permeability (Sharpton et al., 2017). Sharpton et al. (2017) also reported that the glycosaminoglycan degradation pathway increased as colitis progressed in patients with IBD. In addition, these GAGs were depleted in patients’ intestines (Murch et al., 1993), and GAG metabolism was considered relevant to intestinal colonization (Benjdia et al., 2011; Ulmer et al., 2014). Our identification of pathways may contribute to novel hypotheses determining the cause-and-effect between the gut microbiota and IBD.

Several studies have shown contradictory results. They found that *A. muciniphila* was strongly associated with higher rates of tumorigenesis in colorectal cancer, and some mucin-degradation genes were positively correlated with cancer incidence (Baxter et al., 2014). Colonic anaerobic bacteria could ferment ingested carbohydrates and proteins into SCFAs (Cummings, 1981). SCFAs are known as the main energy source for colonocytes, and a deficiency of this energy source may cause the onset of colitis (Roediger, 1980). The strong correlation between *A. muciniphila* and colorectal cancer might be due to the energy dependence. In addition, several studies have found that excessive mucin degradation may aggravate colitis by allowing increased microbial access to the mucus layer (Ganesh et al., 2013; Seregin et al., 2017). However, a previous study has demonstrated that the mucin degradation properties of *A. muciniphila* could provide nutrition for other gut commensals and produce SCFAs (Wu et al., 2017), which are beneficial to the host as we discussed above. In our study, we found that *A. muciniphila* could exert a beneficial role through a combination of different factors.

More importantly, fecal microbiota transplant (FMT) and probiotics are being increasingly used to treat UC, but their use is controversial because of their uncertain efficacy. FMT is recognized as a novel therapy of UC, as it may alter the gut microbial dysbiosis (Sood et al., 2019). Four randomized controlled trials (RCTs) along with several cases have showed the efficacy of FMT to induce remission in active UC patients (Angelberger et al., 2013; Kump et al., 2013; Kunde et al., 2013; Cui et al., 2015; Moayyedi et al., 2015; Ren et al., 2015; Rossen et al., 2015; Suskind et al., 2015; Wei et al., 2015; Paramsothy et al., 2017; Costello et al., 2019). A meta-analysis including 1491 patients with UC (from 18 placebo-controlled and active treatment-controlled studies) found that probiotics were beneficial for the induction of remission in patients with UC (Asto et al., 2019). Recently, a proof-of-concept prospective study unequivocally showed that long-term (3 months) administration of *A. muciniphila* (10^10^ cells per day) was safe and verified the feasibility and tolerance of *A. muciniphila* supplementation in humans (Depommier et al., 2019). *A. muciniphila* is emerging as a novel protective agent, but the efficacy, reliability and safety of *A. muciniphila* in patients with IBD still need to be determined.

Nevertheless, there are certain limitations to our study. The animal model we used mimics some typical histopathological and certain immunological characteristics of patients with IBD, but the model is not fully representative of the complex features of the disease. Additionally, our study demonstrated a correlation between alleviated colitis and *A. muciniphila*-modified gut microbiota. Other potential mechanisms are needed, and further clinical studies in humans with IBD need to be investigated.

## Conclusion

Overall, we confirmed that *A. muciniphila* administration could alleviate mucosal inflammation either by microbe-host interactions that protect the gut epithelial barrier function and downregulate the levels of related inflammatory cytokines or by improving the microbial community. Our findings show that *A. muciniphila* exerted a protective effect on DSS-induced colitis in mice, indicating that *A. muciniphila* may be a potential probiotic agent for ameliorating colitis.

## Data Availability Statement

The datasets generated for this study can be found in NCBI https://www.ncbi.nlm.nih.gov/sra/?term=PRJNA554980.

## Ethics Statement

The animal study was reviewed and approved by the Local Committee of Animal Use and was performed in accordance with the criteria of “the Animal Care Committee of Zhejiang University School of Medicine”, “Guide for the Care and Use of Laboratory Animals” (NIH publication 86–23 revised 1985). Written informed consent was obtained from the owners for the participation of their animals in this study.

## Author Contributions

XB, WW, and YL designed the experiments. XB and LY analyzed the data. XB wrote the manuscript. All authors reviewed the manuscript.

## Conflict of Interest

The authors declare that the research was conducted in the absence of any commercial or financial relationships that could be construed as a potential conflict of interest.

## Footnotes

1 https://www.arb-silva.de/

## Supplementary Material

FIGURE S1 Bar charts represents the serum cytokine levels of IL-1α, TNF-α, IL6, IL12A, IL12B, lL17, eotaxin, G-CSF, KC, MIP-1A, RANTES, and IL10 among the groups. ^∗^*P* < 0.05; ^∗∗^*P* < 0.01; ^∗∗∗^*P* < 0.001; ^****^*P* < 0.0001 by *post hoc* one-way ANOVA.

FIGURE S2 *Akkermansia muciniphila* reshaped the gut microbiota community. The rarefaction curves of three groups.

FIGURE S3 PISRUST results of predicted metabolic pathways in the AKK group (Green) and DP group (Orange).

TABLE S1 Specific primers used for the RT-PCR analyses.

TABLE S2 MRPP test used to analyze the β diversity in fecal analysis.
