# Supplementary figures and images for: Administration of Akkermansia muciniphila Ameliorates Dextran Sulfate Sodium-Induced Ulcerative Colitis in Mice
Source: Front Microbiol. 2019 Oct 1;10:2259. doi: 10.3389/fmicb.2019.02259 (PMC6779789; doi:10.3389/fmicb.2019.02259)

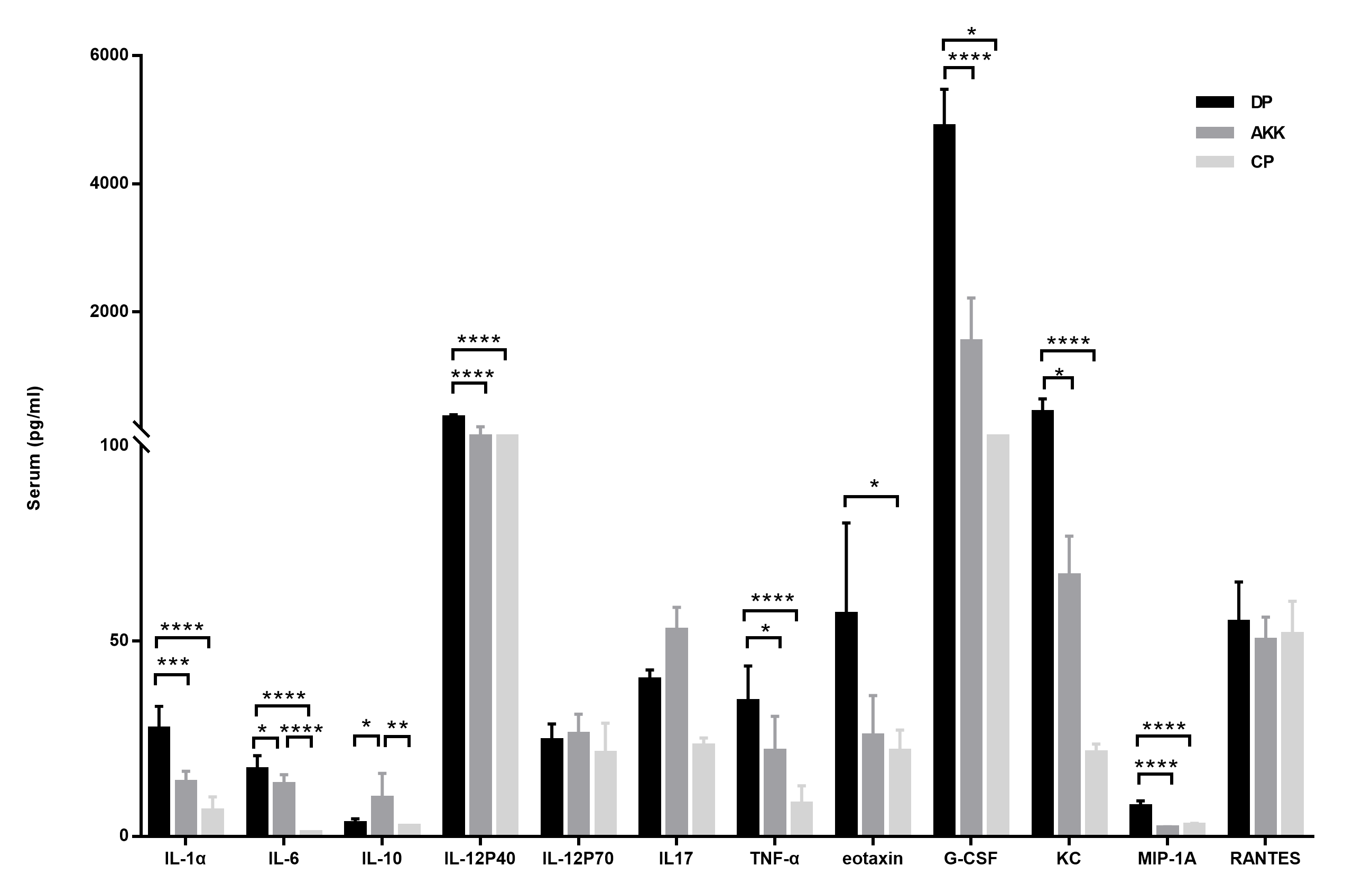

Supplement: FIGURE S1 — Bar charts represents the serum cytokine levels of IL-1α, TNF-α, IL6, IL12A, IL12B, lL17, eotaxin, G-CSF, KC, MIP-1A, RANTES, and IL10 among the groups. ∗P < 0.05; ∗∗P < 0.01; ∗∗∗P < 0.001; ****P < 0.0001 by post hoc one-way ANOVA. [file Image_1.TIF]

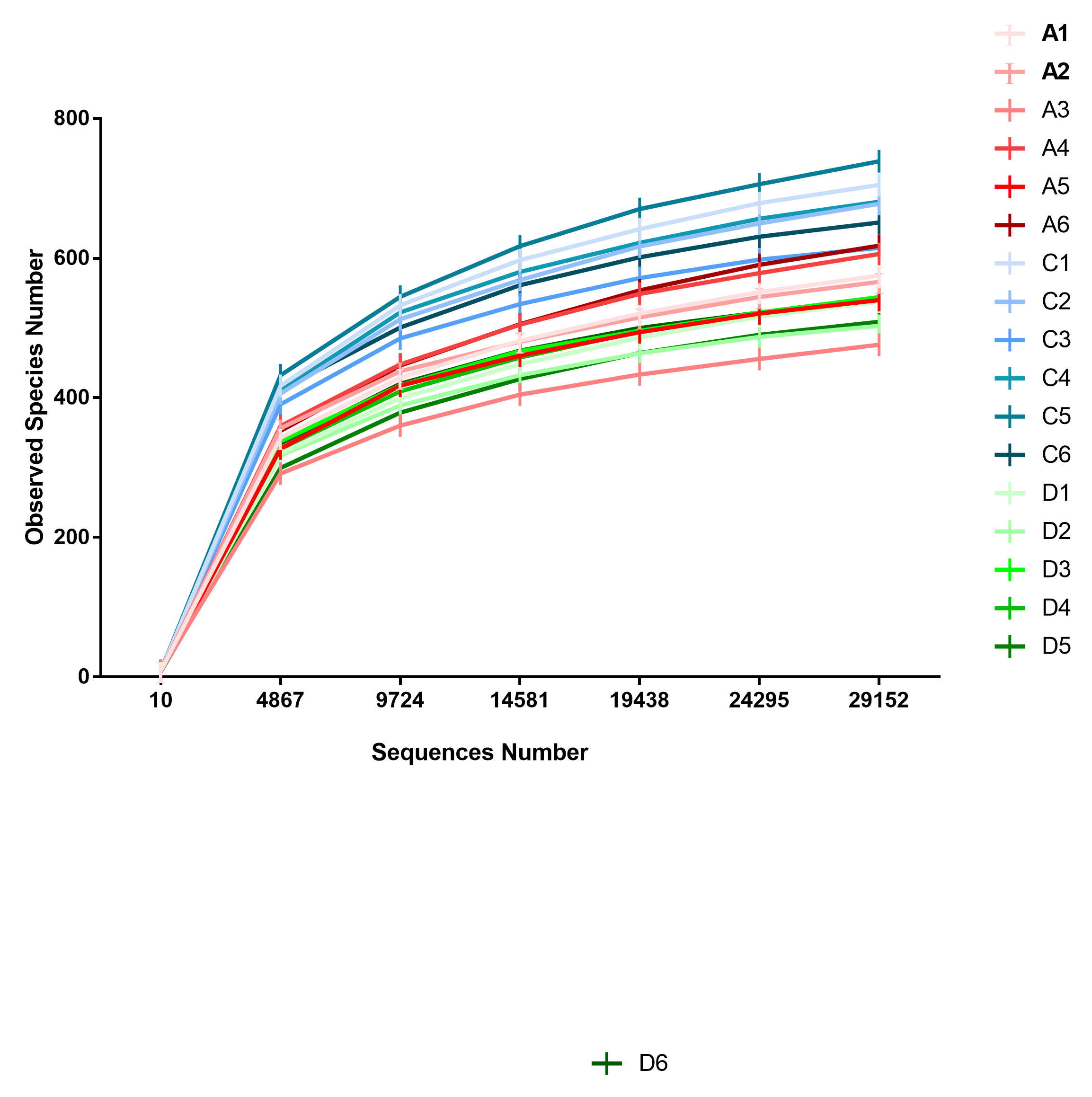

Supplement: FIGURE S2 — Akkermansia muciniphila reshaped the gut microbiota community. The rarefaction curves of three groups. [file Image_2.TIF]

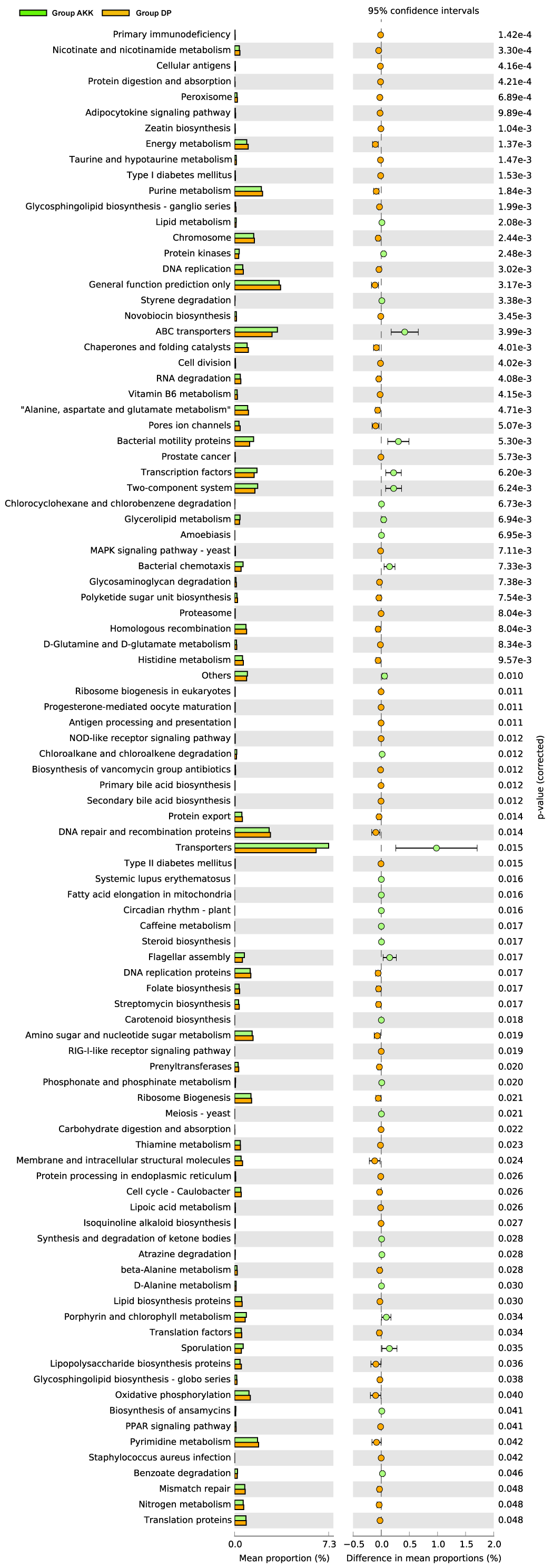

Supplement: FIGURE S3 — PISRUST results of predicted metabolic pathways in the AKK group (Green) and DP group (Orange). [file Image_3.TIF]
