# Supplementary material for: Administration of Akkermansia muciniphila Ameliorates Dextran Sulfate Sodium-Induced Ulcerative Colitis in Mice
Source: Front Microbiol. 2019 Oct 1;10:2259. doi: 10.3389/fmicb.2019.02259 (PMC6779789; doi:10.3389/fmicb.2019.02259)
Supplement: TABLE S1 — Specific primers used for the RT-PCR analyses. [file Table_1.DOCX]

Supplementary Table S1 Specific primers used for the RT-PCR analyses.

| **Gene** | **Forward Sequence (5 ' - 3 ' )** | **Reverse Sequence (5' - 3 ' )** |
| --- | --- | --- |
| **b**-actin | AGTGTGACGTTGACATCCGT | GCAGCTCAGTAACAGTCCGC |
| IL12A | CTGTGCCTTGGTAGCATCTATG | GCAGAGTCTCGCCATTATGATTC |
| MIP-1A | TTCTCTGTACCATGACACTCTGC | CGTGGAATCTTCCGGCTGTAG |
| IL10 | CTTACTGACTGGCATGAGGATCA | GCAGCTCTAGGAGCATGTGG |
| IL6 | TAGTCCTTCCTACCCCAATTTCC | TTGGTCCTTAGCCACTCCTTC |
| IFN**γ** | TCAAGTGGCATAGATGTGGAAGAA | TGGCTCTGCAGGATTTTCATG |
| TNF**α** | AGGCACTCCCCCAAAAGAT | CAGTAGACAGAAGAGCGTGGTG |
| IL1**α** | CGAAGACTACAGTTCTGCCATT | AAACTTCTGCCTGACGAGCTT |
| CB1 | CTGATGTTCTGGATCGGAGTC | TCTGAGGTGTGAATGATGATGC |
| CB2 | TGACAAATGACACCCAGTCTTCT | ACTGCTCAGGATCATGTACTCCTT |
| occludin | TTCCTCTGACCTTGAGTGTGG | CTCTTGCCCTTTCCTGCTTT |
| claudin-4 | GTCCTGGGAATCTCCTTGGC | TCTGTGCCGTGACGATGTTG |
| ZO-1 | GCCGCTAAGAGCACAGCAA | GCCCTCCTTTTAACACATCAGA |

**Abbreviation: IL,interleukin; MIP, macrophage inflammatory protein；TNF-α, tumour necrosis factor alpha; IFNγ, Interferonγ; CB1, cannabinoid receptor 1; CB2, cannabinoid receptor 2; ZO-1,** Zonula occludens-1/Tight junction protein1**;**

Supplementary Table S2 MRPP test used to analyse the β diversity in fecal analysis

| Group | A | observed-delta | expected-delta | Significance |
| --- | --- | --- | --- | --- |
| CP-DP | 0.2086 | 0.4443 | 0.5614 | 0.002 |
| AKK-CP | 0.2521 | 0.4252 | 0.5685 | 0.003 |
| AKK-DP | 0.05727 | 0.4991 | 0.5295 | 0.002 |

MRPP: Multi ResponsePermutation Procedure; Observe Delta: intragroup difference; Expect delta: intergroup difference; A>0: intergroup difference>intragroup difference; A<0: intragroup difference>intergroup difference; Significance<0.05: significant intergroup different.
